# CsPbCl_3_ → CsPbI_3_ Exchange in Perovskite Nanocrystals Proceeds through a Jump-the-Gap Reaction Mechanism

**DOI:** 10.1021/jacs.3c06214

**Published:** 2023-09-11

**Authors:** Nikolaos Livakas, Stefano Toso, Yurii P. Ivanov, Tisita Das, Sudip Chakraborty, Giorgio Divitini, Liberato Manna

**Affiliations:** † Nanochemistry, Istituto Italiano di Tecnologia, Via Morego 30, 16163 Genova, Italy; ‡ Dipartimento di Chimica e Chimica Industriale, Università di Genova, 16146 Genova, Italy; § Electron Spectroscopy and Nanoscopy, Istituto Italiano di Tecnologia, Via Morego 30, 16163 Genova, Italy; ∥ Materials Theory for Energy Scavenging (MATES) Lab, Department of Physics, Harish-Chandra Research Institute (HRI), A CI of Homi Bhabha National Institute (HBNI), Chhatnag Road, Jhunsi, Prayagraj 211019, India

## Abstract

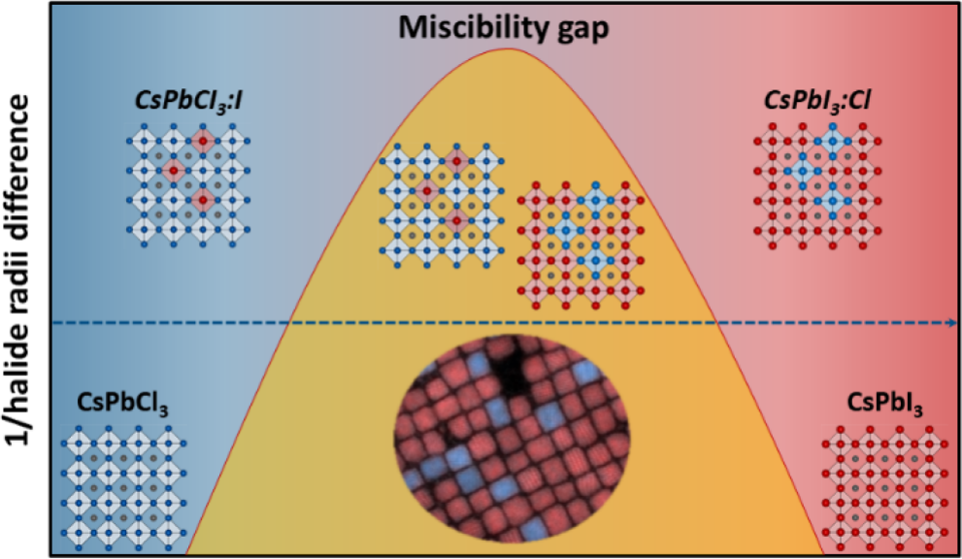

Halide exchange is a popular strategy to tune the properties of CsPbX_3_ nanocrystals after synthesis. However, while Cl → Br and Br → I exchanges proceed through the formation of stable mixed-halide nanocrystals, the Cl ⇌ I exchange is more elusive. Indeed, the large size difference between chloride and iodide ions causes a miscibility gap in the CsPbCl_3_–CsPbI_3_ system, preventing the isolation of stable CsPb(Cl_*x*_I_1–*x*_)_3_ nanocrystals. Yet, previous works have claimed that a full CsPbCl_3_ → CsPbI_3_ exchange can be achieved. Even more interestingly, interrupting the exchange prematurely yields a mixture of CsPbCl_3_ and CsPbI_3_ nanocrystals that coexist without undergoing further transformation. Here, we investigate the reaction mechanism of CsPbCl_3_ → CsPbI_3_ exchange in nanocrystals. We show that the reaction proceeds through the early formation of iodide-doped CsPbCl_3_ nanocrystals covered by a monolayer shell of CsI. These nanocrystals then leap over the miscibility gap between CsPbCl_3_ and CsPbI_3_ by briefly transitioning to short-lived and nonrecoverable CsPb(Cl_*x*_I_1–*x*_)_3_ nanocrystals, which quickly expel the excess chloride and turn into the chloride-doped CsPbI_3_ nanocrystals found in the final product.

## Introduction

All-inorganic lead halide perovskite nanocrystals (LHP NCs) with the composition CsPbX_3_ (X = Cl, Br, I) are widely investigated due to their high photoluminescence quantum yield (PLQY) and narrow emission bandwidth covering the whole visible spectral range.^1,2^ These properties, together with a remarkable tolerance to surface defects,^3,4^ render LHP NCs promising candidates for light-emitting applications.^5−9^ Emission tunability in such materials is typically accomplished by halide alloying,^1,10−12^ obtained either directly during the synthesis or through postsynthetic halide-exchange reactions.^13−16^ Such postsynthetic transformations have been investigated on thin films^13^ even before the advent of NCs and were later studied extensively on nanoparticles in order to establish the mechanism of exchange^17−27^ and suppress it when not desired.^23,24,28^

Thanks to these efforts, Cl–Br and Br–I exchanges are now well understood. However, the exchange between Cl and I anions is not. The very first reported attempts at such exchange, performed by exposing a MAPbCl_3_ film to a solution of MAI,^13^ resulted in the formation of distinct domains of MAPbCl_3_ and MAPbI_3_ instead of the anticipated MAPb(Cl–I)_3_ alloy [MA = methylammonium]. Experiments later performed on colloidal NCs found that, depending on reaction conditions, a direct Cl–I exchange can either lead to the dissolution of NCs^14^ or slowly proceed to a full exchange.^15^ Unfortunately, in this second case, no information on the intermediate steps of the exchange was collected, although a much slower kinetics than that of Cl–Br and Br–I exchanges suggests that the reaction does not proceed through a simple and gradual halide replacement in the NC structure. Later works further complicated the riddle by establishing that Cl^–^ ions can indeed dope CsPbI_3_ NCs, but only in small percentages due to the large difference in the ionic radii of Cl^–^ and I^–^ anions, as the induced strain results in the instability of Cl/I alloys above a certain doping threshold.^29,30^ Even more interestingly, recent reports revealed that CsPbCl_3_ and CsPbI_3_ NCs mixed together can coexist without undergoing any apparent reaction, in stark contrast with the almost immediate exchange taking place between mixtures of CsPbCl_3_–CsPbBr_3_ and CsPbBr_3_–CsPbI_3_ NCs.^24,30,31^

In this work, we provide a clearer view of the CsPbCl_3_ → CsPbI_3_ halide exchange in NCs by focusing on its reaction mechanism and involved intermediates. In agreement with early reports on thin films,^13^ we found that the exchange proceeds through the formation of two separate populations of CsPbCl_3_ and CsPbI_3_ NCs, whose relative fractions vary over the course of the reaction. An in-depth investigation revealed that these populations do incorporate a small fraction of the opposite halide but with remarkably low saturation thresholds (a few %). This forces the reaction to proceed through a sudden leap across the CsPbCl_3_–CsPbI_3_ miscibility gap, where Cl-doped CsPbI_3_ NCs suddenly turn into I-doped CsPbCl_3_ NCs by transitioning through short-lived and barely observable CsPb(Cl_*x*_I_1–*x*_)_3_ alloyed NCs. Interestingly, the poor solubility of I in CsPbCl_3_, together with the need for allocating the excess iodide in solution, also caused the accumulation of CsI on the surface of CsPbCl_3_ NCs, resulting in a passivation layer resembling the structure of the all-inorganic Ruddlesden–Popper phase Cs_2_PbCl_2_I_2_. Remarkably, such a CsI layer allows iodide to be stably present on the surface of the CsPbCl_3_ NCs without straining the underlying NC lattice, which would instead be the case for a CsPbI_3_ layer.

## Results and Discussion

### Steady-State Investigation of the Cl → I Halide Exchange

**Figure 1:**
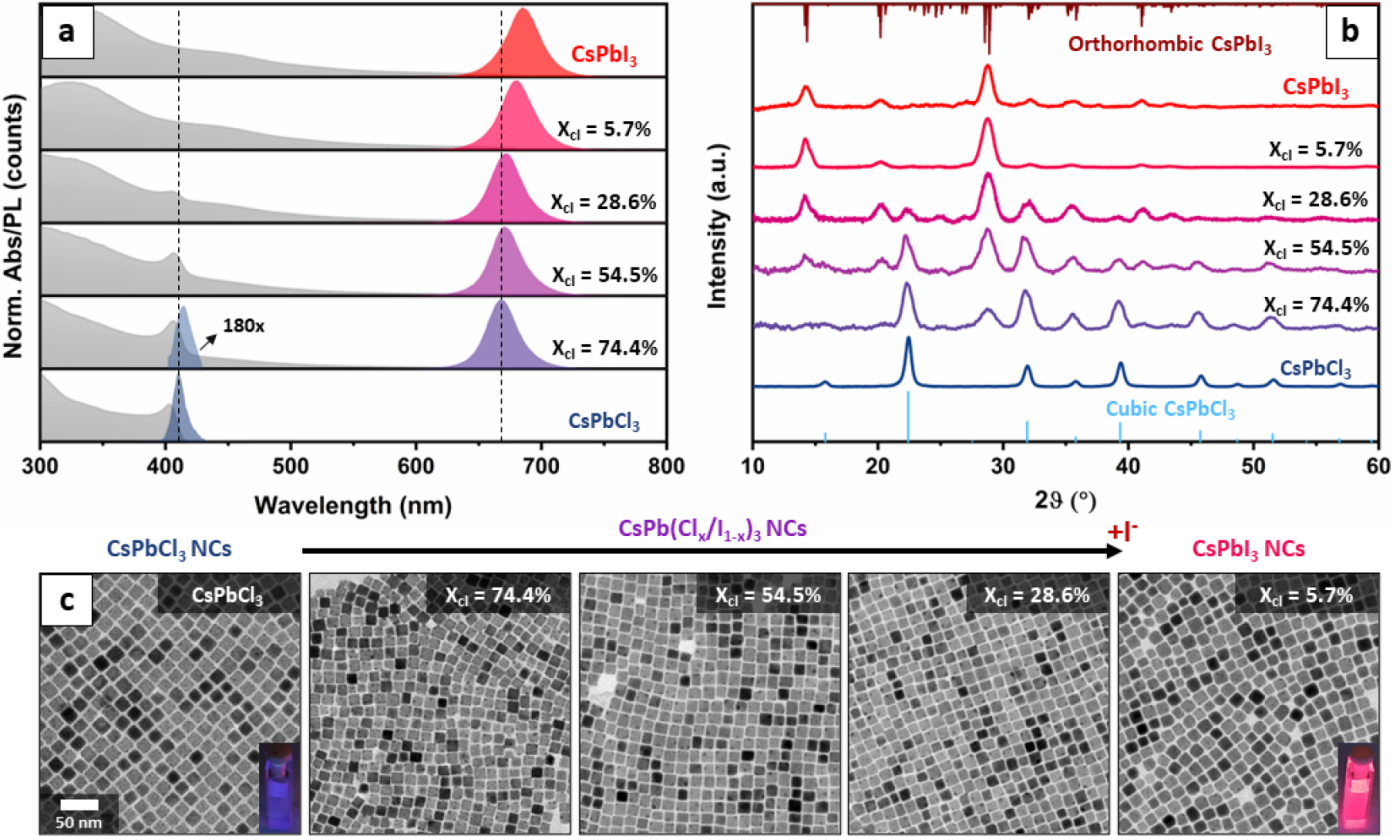
Steady-state investigation of the Cl → I halide exchange. (a) ABS (gray) and PL (colored) spectra of pure-halide CsPbCl_3_ (bottom spectra) and CsPbI_3_ NCs (top spectra) and of CsPbCl_3_ NCs exchanged with increasing amounts of PbI_2_. The reaction first produces a suppression of the CsPbCl_3_ PL signal, followed by the appearance of the CsPbI_3_ ABS and PL signals. A spectral shift is observed in the PL signal of reacted NCs with respect to the pure-halide references, suggesting a certain degree of halide alloying. (b) XRD patterns of the same samples, confirming the coexistence of the two phases during the exchange and a progressive evolution of their relative amounts. (c) TEM images collected on NCs as synthesized (left) and at different stages of the exchange (other panels), showing that the reaction has little impact over the NCs’ morphology.

**Figure 2:**
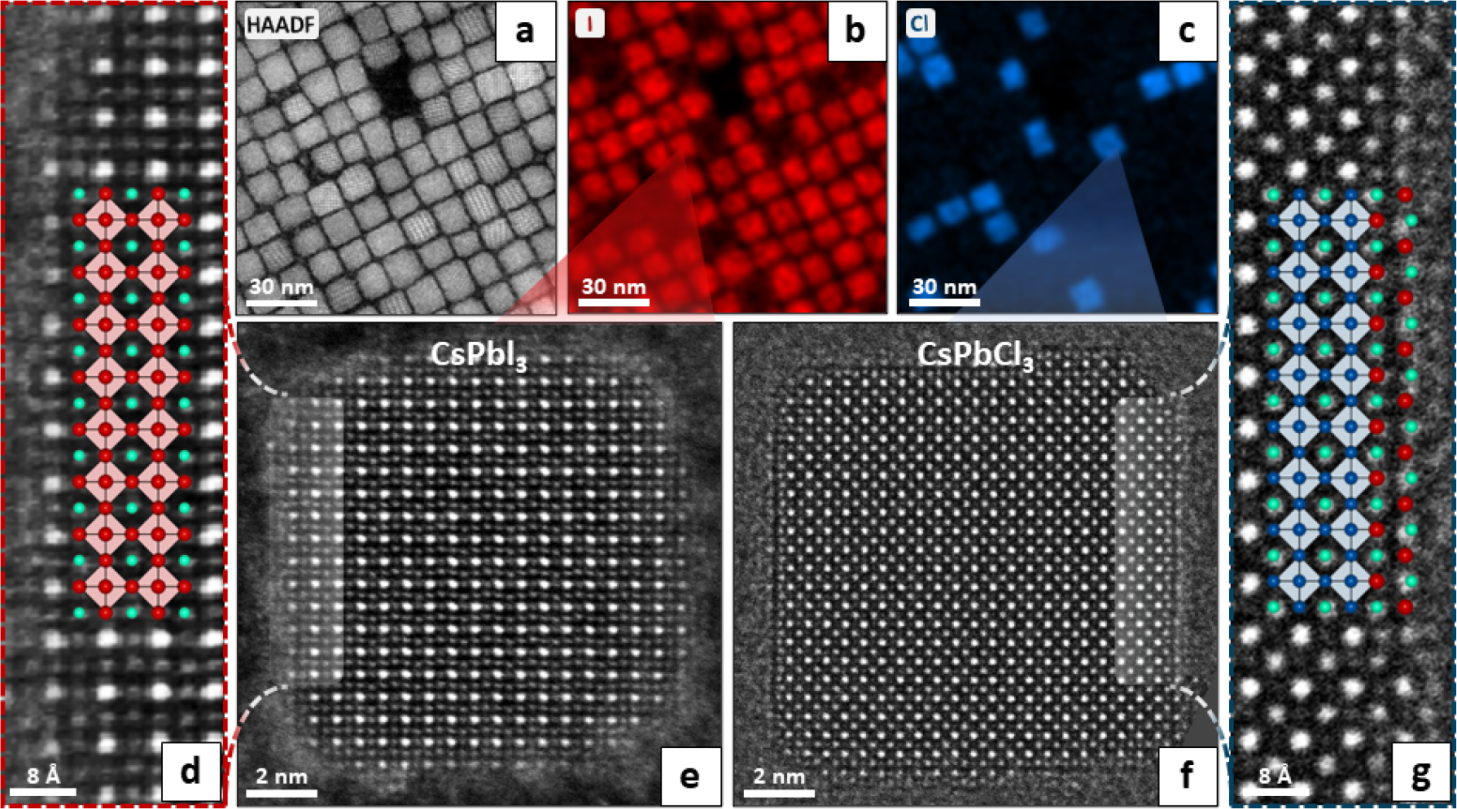
Structure and composition of exchanged NCs. (a) HAADF-STEM image of exchanged NCs (sample labeled *x*_Cl_ = 28.6% in Figure 1). (b, c) STEM-EDX elemental maps for chloride (blue) and iodide (red), showing the coexistence of two separate NC populations. (e, f) Atomic-resolution representative images of a CsPbCl_3_ NC (right) and of a CsPbI_3_ NC (left), showing that the exchange process does not affect the crystallinity of particles. We note that the HAADF-STEM images were not acquired on the same exact NCs shown in panels b,c, and the zoom cones are only meant as a graphic element to improve clarity. (d) Enlarged view of the CsPbI_3_ NC surface (Cs^+^ = green, I^–^ = red, and [PbI_6_]^4–^ octahedra = light red). (g) Enlarged view of the CsPbCl_3_ NC surface, highlighting the presence of a Cs–I surface plane inconsistent with the structure of bulk CsPbCl_3_ (Cs^+^ = green, Cl^–^ = blue, I^–^ = red, and [PbCl_6_]^4–^ octahedra = light blue). An analogous behavior was observed for the rest of the partial exchange cases, i.e., for *x*_Cl_ = 74.4% and 54.5% (Figure S8).

The postsynthetic CsPbCl_3_ → CsPbI_3_ halide exchange reaction was investigated on Cs-oleate-capped CsPbCl_3_ NCs with an edge length of ∼14 nm (Figures 1c and Supporting Information [SI], Figures S1 and S2). These NCs were prepared following our previously reported protocol, which offers good size tunability and phase purity (see SI and Experimental Section for details).^32^ The halide exchange reactions were performed using PbI_2_ dissolved in a mixture of oleylamine (OLA) and oleic acid (OA) as a halide source. During the experiments, the NCs were reacted with increasing amounts of PbI_2_ for a constant time of 10 min, enough to reach equilibrium conditions. The volumes and concentrations used for the reactions are detailed in the Experimental Section and SI presented in Table S1. After the exchange, the final halide ratios in the samples were determined by scanning electron microscopy-energy-dispersive X-ray spectroscopy (SEM-EDX) and fell in the range *x*_Cl_ = [Cl]/[Cl] + [I] % = 100% (pristine CsPbCl_3_) to *x*_Cl_ = 5.7%. As demonstrated by steady-state absorption (ABS) and photoluminescence (PL) spectroscopy (Figure 1a) and X-ray diffraction (XRD, Figure 1b) measurements, increasing the amount of added PbI_2_ induced a progressive anion exchange. Specifically, XRD indicates that the addition of PbI_2_ above a certain threshold caused the formation of CsPbI_3_ NCs alongside the initial CsPbCl_3_ NCs and that the relative fraction of CsPbI_3_ grew as the iodide content in the sample increased. This is consistent with the appearance of a PL peak in the red region of the spectrum that became progressively closer to the band-edge emission of pure CsPbI_3_ (purple-red in Figure 1a) and with the significant quenching of the CsPbCl_3_ emission (blue in Figure 1a). Such quenching is likely due to the formation of a CsI layer on the surface of CsPbCl_3_ NCs, which is discussed later in the context of Figure 2. Notably, transmission electron microscopy (TEM) shows that the exchange procedure has little impact on the NCs’ morphology (Figures 1c and S2). As a control, we verified that reactions performed on NCs with a different capping agent (i.e., dimethyl didodecylammonium chloride, DDACl) and by using different halide precursors (i.e., benzoyl iodide and trimethylsilyl iodide) produced comparable results (Figures S3–S5). The only difference was some morphological damage to the NCs when halide sources other than PbI_2_ were used, which was, however, mild in the case of DDACl-capped NCs. As no major differences were found, we opted to focus exclusively on Cs-oleate-capped NCs reacted with PbI_2_ for further characterizations.

An in-depth analysis of the XRD data collected on samples with different halide ratios (Figure 1 and Figure S6) revealed that the pseudocubic lattice parameter of both phases (CsPbCl_3_ and CsPbI_3_) expanded as the iodide content of the sample increased (CsPbCl_3_: 5.60 → 5.64 Å; CsPbI_3_: 6.19 → 6.22 Å; see Figure S7 and Table S2). This clearly indicates that both compounds could incorporate a small fraction of the opposite halide, which depended on the total amount of PbI_2_ added for the reaction. By applying Vegard’s law,^33^ we estimated the miscibility thresholds to be ∼6% I in CsPbCl_3_ and ∼4% Cl in CsPbI_3_, measured on the last detectable signal of CsPbCl_3_ and the first detectable signal of CsPbI_3_, respectively. This is consistent with the subtle shift of the emission spectra observed for exchanged NCs with respect to the corresponding pure-halide CsPbCl_3_ and CsPbI_3_ NCs (Figure 1a). We remark that these changes cannot be ascribed to quantum confinement effects, as all samples had similar average size (∼14 nm), which was above the confinement regime for lead halide perovskites.^1,34^

### HAADF-STEM Characterization of Exchanged NCs

**Figure 3:**
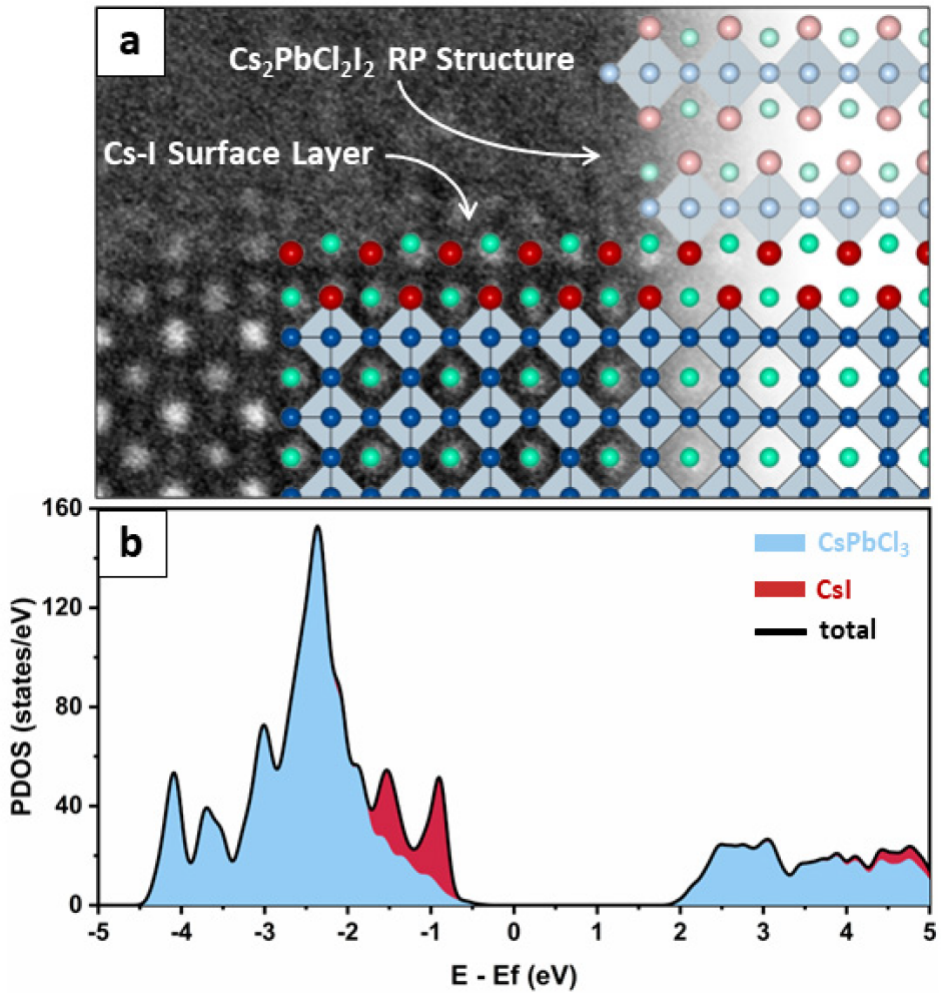
Ruddlesden–Popper-like CsI termination of CsPbCl_3_:I NCs. (a) Left: enlarged view of a CsPbCl_3_ NC surface, with overlay of the corresponding atomistic model. Right: idealized CsPbCl_3_/Cs_2_PbCl_2_I_2_ epitaxial interface, showing that the CsI layer forming on the NC surface is identical to the Cs–I plane found in the all-inorganic Ruddlesden–Popper phase.^35^ Such behavior is consistent with the Cs^+^ sublattice preservation principle often observed in epitaxial interfaces formed by Cs–Pb–X compounds.^37^ (b) Integrated DOS calculated for the composite (CsPbCl_3_ including the CsI shell). The contribution of the CsPbCl_3_ NC volume to the DOS is depicted in blue, whereas red represents the contribution of the CsI layer to the composite DOS. Being close to the band edge, these additional states likely act as nonradiative trap states.

The coexistence of CsPbCl_3_ and CsPbI_3_ NCs during the exchange was further confirmed by high-resolution scanning TEM–energy dispersive X-ray spectroscopy (HRSTEM-EDX, Figure 2a–c), where the Cl and I elemental mapping revealed the presence of two distinct populations of NCs. Interestingly, although little to no chloride was detected in the CsPbI_3_ NCs, some iodide was visible in or around the CsPbCl_3_ NCs. This apparently contrasts with the XRD results that suggested comparable solubility thresholds for Cl in CsPbI_3_ and vice versa. However, atomic resolution images of the same particles (Figure 2d–g, see also Figures S9 and S10) showed that such excess is not found in the volume of the CsPbCl_3_ NCs, where it would have been detected by XRD through lattice expansion but rather on their surface in the form of a CsI layer. Remarkably, such a configuration allows the CsPbCl_3_ NCs to capture some iodide from the solution without inducing strain in the lattice. Indeed, due to the large difference in the lattice parameters of CsPbCl_3_ and CsPbI_3_ (5.60 Å vs 6.22 Å), alloying large amounts of iodide within the volume of CsPbCl_3_ NCs would be unfavorable. However, the observed CsI layers are structurally analogous to the corrugated Cs–I planes found in the all-inorganic Ruddlesden–Popper phase Cs_2_PbCl_2_I_2_ (Figure 2g, Figure 3a), whose lattice parameter is much closer to that of CsPbCl_3_ (5.65 Å vs 5.60 Å).^35,36^ Therefore, the strain induced by such a layer on the surface of CsPbCl_3_ NCs is negligible, while its formation is favored by the large excess of free iodide present in solution. We remark that a similar CsCl termination is not viable for CsPbI_3_ NCs, as surface layers with inverted halide positions resemble no known stable structures. Indeed, CsPbI_3_ NCs at higher magnification show no evidence of a CsCl coating (Figure 2d).

Interestingly, the calculated electronic structure of CsI-coated CsPbCl_3_ NCs (Figure 3b) features a peak in the density of states (DOS) close to the CsPbCl_3_ valence band edge, which is attributed to the I^–^ anions of the CsI surface layer.^35^ As the photoluminescence quantum yield reported for Cs_2_PbCl_2_I_2_ NCs is extremely low,^36^ we hypothesize by analogy that such states act as traps and contribute to the quenching of CsPbCl_3_ photoluminescence observed in Figure 1a.

### In Situ Investigation of the Exchange Kinetics

**Figure 4:**
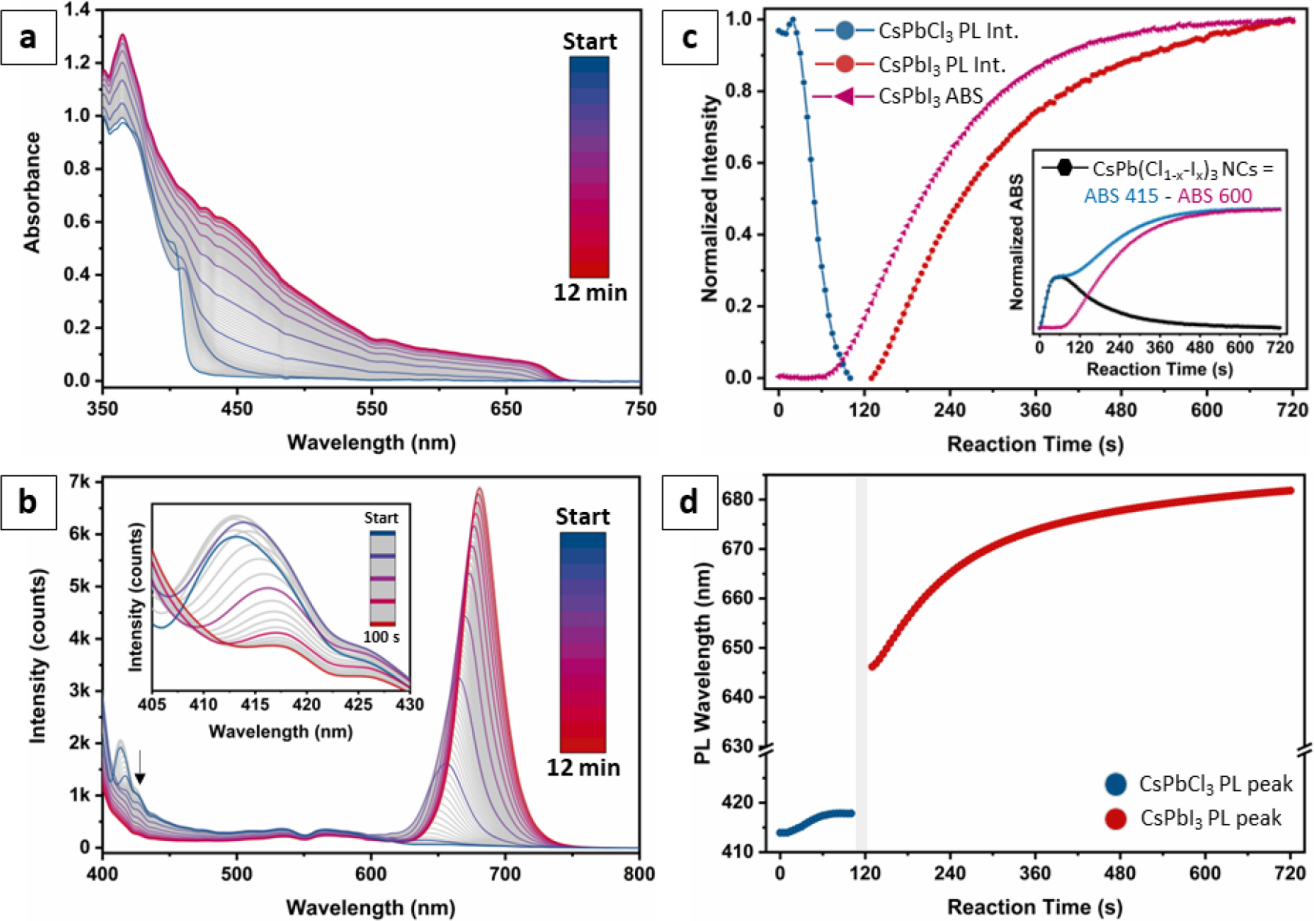
Exchange reaction followed by in situ spectroscopy. (a) In situ ABS spectra collected every 5 s for a total of 12 min, starting right after the injection of the PbI_2_ solution in a sample of pristine CsPbCl_3_ NCs. Colored spectra were collected 1 min apart. (b) In situ PL spectra collected in the same reaction conditions as for panel (a), using a 365 nm LED as the excitation source. The nonflat background and artifacts visible in the 400–625 nm spectral range are part of the emission tail of the lamp. Inset: magnification of the high-energy part of the PL spectra during the first ∼2 min of the reaction, showing a sudden quenching of the CsPbCl_3_ PL. (c) Normalized temporal evolutions of CsPbI_3_ ABS (tracked at 600 nm, pink line), CsPbI_3_ PL (peak amplitude, red line), and CsPbCl_3_ PL (peak amplitude, blue line). The CsPbCl_3_ ABS could not be tracked due to the spectral overlap with the ABS band of the PbI_2_ precursor (Figure S12) and of CsPbI_3_ NCs. Inset: ABS evolution of the intermediate CsPb(Cl_*x*_I_1–*x*_)_3_ NCs (black line), obtained as the difference between normalized ABS signals at 415 nm (tail + CsPbI_3_, blue line) and at 600 nm (CsPbI_3_ only, pink line). (d) Evolution of the PL peak position for CsPbCl_3_ (blue) and CsPbI_3_ (red). The region in gray indicates the time range during which no PL was observed from either material.

To complete our understanding of the Cl → I exchange, we investigated the progress of the reaction by in situ ABS and PL spectroscopy (Figure 4). Both experiments were performed by adding a known amount of the PbI_2_ precursor to solutions of CsPbCl_3_ NCs and tracking the subsequent evolution of spectra with an immersion probe. The reaction conditions were chosen to ensure that a full exchange, with no residual CsPbCl_3_ NCs left, could be achieved at the end of the process. Details are provided in the Experimental Section. In situ ABS spectroscopy (Figure 4a) showed the early formation of an absorption tail at energies right below the excitonic peak of CsPbCl_3_ NCs (405 nm), which started growing immediately after the addition of PbI_2_ and reached its maximum intensity at ∼1 min, after which it started fading (Figure 4c, inset). Crucially, its appearance coincided with the smoothing and red-shift of the CsPbCl_3_ excitonic peak. At approximately the same time, we first detected the broad absorption profile of CsPbI_3_ NCs, which during the course of the reaction grew at the expense of both the CsPbCl_3_ band edge and its absorption tail (Figure 4c). This suggests that such a tail is related to an iodide-containing species, which is converted into CsPbI_3_ NCs as the reaction proceeds. Based on these premises, we attributed both the red-shift of the CsPbCl_3_ NC band edge and the appearance of the absorption tail shown in the inset of Figure 4c to the early formation of alloyed CsPb(Cl_*x*_I_1–*x*_)_3_ NCs, which is energetically disadvantaged but likely kinetically favored.

The in situ PL spectra are in line with the observations above (Figure 4b). In the first ∼2 min after the injection of PbI_2_, the PL signal of CsPbCl_3_ NCs experienced a small burst, likely due to the replacement of the original oleate termination with the oleylammonium present in the PbI_2_ solution, followed by a swift drop and its eventual extinction (Figure 4b, inset). Such a dynamic preceded the nucleation of CsPbI_3_ and coincided with the tail attributed to CsPb(Cl_*x*_I_1–*x*_)_3_ NCs reaching their peak intensity. This indicates that the quenching of the PL was not due to the disruption of NCs, but rather to the introduction of trap states, which we ascribe to the formation of the Cs–I surface layers. Indeed, it is worth noting that previous reports observed the formation of Ruddlesden–Popper-like stacking faults in the volume of CsPbBr_3_ NCs co-doped with both Cl and I, which was correlated to a severe PL quenching.^36^ These defects are structurally very similar to the Cs–I layers we observed on the surface of CsPbCl_3_ NCs (Figure 3), and it is likely that in the early stages of the exchange process similar stacking faults might have temporarily formed within the volume of our NCs as well. Conversely, we can exclude that the PL quenching is due to the formation of halide vacancies or to the loss of surface ligands because the transformation is triggered by adding a solution containing an excess of both iodide and surfactants, which should instead passivate the NCs’ surface better. Indeed, adding only ligands to CsPbCl_3_ NCs (Figure S11) resulted as expected in an increase of their PL intensity.

As the reaction proceeded, the PL signal of CsPbI_3_ NCs became visible (*t* ∼ 2.5 min, Figure 4b). Notably, the PL peak of both CsPbCl_3_ and CsPbI_3_ NCs experienced a progressive red-shift as the exchange advanced (Figure 4d). In the case of CsPbCl_3_, this is explained by the incorporation of iodide, as discussed above. In the case of CsPbI_3_ instead, it is likely that the PL signal became detectable as the alloyed CsPb(Cl_*x*_I_1–*x*_)_3_ NCs, metastable and not brightly emissive, progressively released the excess Cl in the reaction medium and saw their emission restored. This hypothesis would also explain the temporal offset between the time evolution curves of CsPbI_3_ ABS and PL (Figure 4c), as the ABS signal could be detected even when the PL was still quenched. We also remark that at the end of the reaction the PL of CsPbI_3_ NCs did not match the spectral position characteristic of as-synthesized CsPbI_3_ NCs of comparable sizes. This, together with the XRD analysis discussed above, clearly indicates that there is residual solubility of Cl in CsPbI_3_ and vice versa.

These observations, combined with the steady-state studies summarized in Figure 1, suggest a jump-the-gap exchange mechanism, where the pristine CsPbCl_3_ NCs quickly transition to CsPbI_3_ NCs through the formation of metastable and short-lived CsPb(Cl_*x*_I_1–*x*_)_3_ alloyed NCs. This is supported by the discontinuity of the PL spectral positions made clear by Figures 1a and 4b,d, and by the temporary appearance of a short-lived absorption feature between the band edges of CsPbCl_3_ and CsPbI_3_, which we attribute to the quick onset and then consumption of the CsPb(Cl_*x*_I_1–*x*_)_3_ intermediates. We remark however that a direct observation of such intermediates under the TEM is likely impossible due to their short life in solution (∼6 min, see inset of Figure 4c) and to their expected tendency to evolve into either CsPbCl_3_ or CsPbI_3_ NCs by expulsion of the excess halide in solution.

### Proposed Reaction Mechanism

**Scheme 1:**
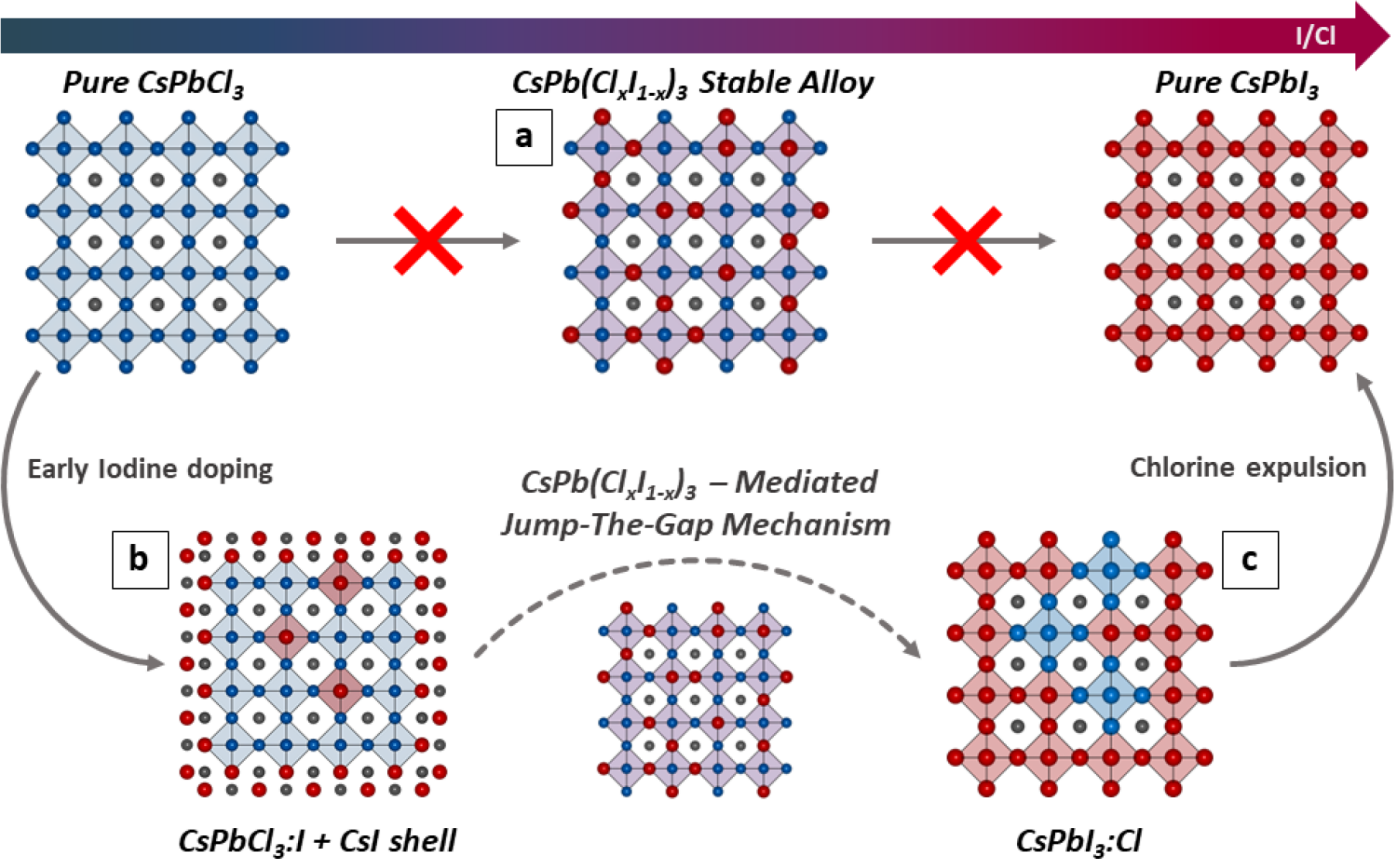
CsPbCl_3_ → CsPbI_3_ Halide Exchange Mechanism Differently from Cl → Br and Br → I exchanges, the CsPbCl_3_ → CsPbI_3_ exchange reaction cannot proceed through stable and recoverable CsPb(Cl–I)_3_ alloyed NCs due to the miscibility gap of these phases (a). Instead, the reaction proceeds through the early formation of I-doped CsPbCl_3_ NCs, which soon hit the saturation limit (b). At this point, the excess of iodide in solution induces two parallel phenomena: the formation of a CsI shell on the surface of CsPbCl_3_ NCs, which act as temporary iodide reservoirs, and (c) the sudden transformation of CsPbCl_3_ NCs into CsPbI_3_ NCs, likely mediated by the temporary formation of metastable and nonrecoverable CsPb(Cl–I)_3_ alloyed NCs. As the reaction proceeds, the CsPbCl_3_ NCs are consumed, and the Cl-doped CsPbI_3_ NC progressively expels the excess chloride back in solution (c), eventually approaching the limit of halide-pure CsPbI_3_ NCs.

The combination of in situ spectroscopies and postreaction structural characterization allows us to outline a mechanistic picture of the Cl → I halide exchange process (Scheme 1). In agreement with previous reports, we confirm that the addition of iodide to a solution of CsPbCl_3_ NCs does not proceed through the formation of stable and recoverable mixed-halide CsPb(Cl_*x*_I_1–*x*_)_3_ NCs, as opposed to Cl → Br and Br → I exchanges (Scheme 1a). Instead, the pristine CsPbCl_3_ NCs quickly incorporate, at first, a small amount of iodide, below their saturation threshold, and turn into I-doped CsPbCl_3_ NCs (Scheme 1b). This is in line with the general behavior of lead halide perovskite NCs, which feature remarkably high halide exchange coefficients. At the same time, part of the iodide concentrates on the surface of CsPbCl_3_ NCs, forming a CsI passivation layer whose structure is reminiscent of the all-inorganic Cs_2_PbCl_2_I_2_ Ruddlesden–Popper phase. Overall, these two processes respectively cause a red-shift of the CsPbCl_3_ absorption edge (iodide incorporation) and a quenching of the NCs PL due to the formation of nonradiative trap states (Cs–I layer formation). However, an increasing fraction of these I-doped CsPbCl_3_ NCs start incorporating iodide above their saturation threshold (estimated to be ∼6% by postreaction XRD analyses), turning into metastable CsPb(Cl_*x*_I_1–*x*_)_3_ alloyed NCs (Scheme 1c). These act as short-lived reaction intermediates that quickly evolve forward, expelling the excess chloride and turning into Cl-doped CsPbI_3_ NCs, or backward, returning to the state of I-doped CsPbCl_3_ NCs. This mechanism explains why both populations can be observed if the reaction is arrested before completion (Figure 1) and represents the main difference with respect to Cl → Br and Br → I exchanges, where the alloyed NCs are stable and can be isolated as a reaction product. If the reaction is allowed to proceed to completion, instead, an always increasing fraction of I-doped CsPbCl_3_ NCs is converted through this mechanism into Cl-doped CsPbI_3_ NCs, until their transformation is complete. At this final stage, the sample is fully composed of Cl-doped CsPbI_3_ NCs, which depending on the amount of iodide available will progressively expel the excess Cl and approach the limit of pure-halide CsPbI_3_ NCs (panel 1e).

We remark here that the coexistence of Cl-doped CsPbI_3_ and I-doped CsPbCl_3_ NCs at equilibrium is fully compatible with a classical phase diagram between two compounds with limited miscibility, where the overall composition of the sample impacts the relative ratio of phases more than their individual compositions (Figure S13). Within the frame of this interpretation, the alloyed CsPb(Cl_*x*_I_1–*x*_)_3_ NCs act as out-of-equilibrium transition states that are functional for the system to leap over the miscibility gap.

## Conclusions

In this work, we explored the direct Cl → I halide exchange reaction in colloidal CsPbCl_3_ NCs. In situ spectroscopy demonstrated that the reaction proceeds through the temporary incorporation of iodide in the volume of pristine CsPbCl_3_ NCs, resulting in the formation of metastable CsPb(Cl_*x*_I_1–*x*_)_3_ NCs. We propose that these act as reaction intermediates that evolve by expelling the excess of one of the two halides, turning either into I-doped CsPbCl_3_ or Cl-doped CsPbI_3_ NCs, where the low doping concentration was probably dictated by the solubility limit of one phase in the other. Depending on the amount of iodide added, the reaction could either proceed to a full exchange or be halted to yield a stable mixture of CsPbCl_3_ and CsPbI_3_ NCs, which corresponds to the thermodynamic equilibrium condition for a partially miscible system and therefore did not react further. This behavior differs from what is observed for Cl → Br and Br → I exchanges, which form stable and continuous solid solutions. Such a difference is attributed to the marked size differences of the Cl and I anions, which results in a miscibility gap in the CsPbCl_3_–CsPbI_3_ equilibrium phase diagram. Our proposed reaction mechanism might serve to guide future experiments on this and similar ion exchange reactions and points to the importance of not isolable, and therefore hard to observe, intermediates in reactions involving colloidal nanocrystals.

In addition, we observed the formation of a Cs–I termination on the surface of treated CsPbCl_3_ NCs, whose structure resembled the structural motifs found in the Cs_2_PbCl_2_I_2_ Ruddlesden–Popper phases. We interpret this as a way for the system of quickly accepting the excess of added iodide without incurring the strain generated by the incorporation of iodide in the NCs’ volume. Although these core–shell architectures are detrimental for the photoluminescence of the CsPbCl_3_ cores, they expand the range of currently known nanoscale heterostructures based on metal halides. Moreover, the idea of covering a halide perovskite core with a binary metal halide can be, in principle, extended to other materials with more promising outcomes in terms of preservation or enhancement in optical properties than for the present CsPbCl_3_/CsI system. The formation of stable CsPbI_3_ phases with low levels of Cl alloying (up to 4% Cl) also represents an interesting way to fine-tune the emission wavelength of CsPbI_3_ nanocrystals.

## Experimental Section

### Chemicals

1-Octadecene (ODE, tech, 90%), oleic acid (OA, tech, 90%), oleylamine (OLA, tech, 70%), iodine (I_2_, 99.8%), lead(II) iodide (PbI_2_, 98%), cesium carbonate (Cs_2_CO_3_, 99%), lead acetate trihydrate (Pb(OAc)_2_·3H_2_O, 99.99%), sodium iodide (NaI, 99.99%), benzoyl chloride (C_6_H_5_COCl, 98%), trimethyliodosilane (TMSI, (CH_3_)_3_SiI 97%), methyl acetate (CH_3_COOCH_3_, anhydrous, 99.5%), toluene (TOL, anhydrous, 99.8%), ethyl acetate (CH_3_COOC_2_H_5_, fumed silica powder (SiO_2_), and hexane (99.8%) were purchased from Sigma-Aldrich. Didodecyldimethylammonium chloride (DDACl, 98.0%) and didodecylamine were purchased from TCI. All reagents were used as received without any further experimental purification.

### Oleylammonium Iodide Precursor Solution

This was made by mixing I_2_ powder (1.5 g, 6 mmol) with OLA (9 mL, 0.027 mol) and ODE (21 mL) in a 40 mL glass vial.^38^ The vial was placed on a hot plate and degassed upon vacuum for 30 min at 110 °C. The resulting solution was cooled down at room temperature and stored under nitrogen.

### Lead Iodide Precursor Solution

This was prepared by mixing PbI_2_ powder (0.922 g, 2 mmol), OA (5 mL), OLAM (5 mL), and ODE (30 mL) in a 100 mL three-neck round-bottom flask. The mixture was dried under vacuum for 30 min at 110 °C. Then, the temperature was raised to 150 °C under nitrogen until the salt completely dissolved. The solution was then cooled to room temperature.

### Benzoyl Iodide Precursor Solution

This was prepared by mixing NaI powder (3 g) and benzoyl chloride (1.4 mL) in a 20 mL amount as reported by Imran et al.^39^ The mixture was stirred at 75 °C on a hot plate overnight. Then, the mixture was cooled to room temperature before adding 3 mL of anhydrous toluene. The liquid product was collected after being filtered with a polytetrafluorethylene membrane filter (0.45 μm pore size). Finally, the solution was diluted by adding 30 mL of toluene.

### Synthesis of CsPbCl_3_ NCs

Colloidal CsPbCl_3_ NCs, with an edge-to-edge size of 14 nm, were synthesized based on a synthetic protocol previously reported by our group, with slight modifications.^32^ Briefly, cesium carbonate (16 mg), lead acetate trihydrate (76 mg), and octadecene (10 mL) were added in a 25 mL three-neck flask and dried under vacuum for 1 h at 115 °C using a Schlenk line. Then, 2.5 mL of a ligand solution (consisting of 1.25 mmol of didodecylamine, 1 mL of anhydrous toluene, and 1.5 mL of degassed OA) was injected into the flask. After complete dissolution (∼20 min) of the salts, the temperature was raised to 140 °C, and a solution of benzoyl chloride (50 μL) in degassed octadecene (500 μL) was swiftly injected. The reaction was quenched by an ice/water bath after 3 min. Then, 8 mL of anhydrous methyl acetate was added to the crude solution, and the NC suspension was centrifuged for 5 min at 6000 rpm. The precipitate was redispersed in 15 mL of toluene and used as a stock solution for anion exchange reactions. The concentration of Pb in the NC stock solution was 6.3 mM, as determined by ICP-OES.

### Ligand Exchange

Ligand exchange reactions on the crude solution of Cs-oleate-capped CsPbCl_3_ NCs with DDACl took place under nitrogen flow.^40^ At first, crude solutions were mixed with a solution containing DDACl in anhydrous toluene (4 mL, 25 mM), and the mixture was stirred for 1 min. Afterward, the NCs were purified by addition of ethyl acetate (25 mL) and centrifugation at 6000 rpm for 10 min. Thereafter, the precipitate was dispersed in a solution of anhydrous toluene and DDACl (4 mL, 2.5 mM), washed with ethyl acetate (12 mL), and recovered by centrifugation. This step is repeated for two additional cycles.

### Synthesis of CsPbI_3_ NCs

Colloidal CsPbI_3_ NCs were synthesized following a previously reported procedure from our group.^38^ Cesium carbonate (16 mg), lead acetate trihydrate (76 mg), OA (0.2 mL), and octadecene (5 mL) were added in a 40 mL glass vial and dried for 1 h at 115 °C. Then, the temperature was raised to 165 °C, and 2 mL of the previously prepared oleylammonium iodide solution was swiftly injected under nitrogen. The reaction was quenched with an ice/water bath after 10 s. Then, 5 mL of anhydrous methyl acetate was added to the crude solution, and the NC suspension was centrifuged for 5 min at 6000 rpm. The precipitate was redispersed in toluene.

### Anion Exchange Reactions

All anion exchange reactions were performed under nitrogen. A 6 mL amount of a CsPbCl_3_ stock solution (see above) was used for each reaction, and different amounts of PbI_2_ solution (ranging from 500 to 6 mL) were added under stirring at room temperature. Afterward, the NCs were collected by centrifugation at 6000 rpm for 5 min and redispersed in toluene for further characterization. Similar procedures were followed when other sources of iodide (benzoyl iodide, TMSI) were used for the halide exchange on both Cs-oleate- and DDACl-capped CsPbCl_3_ NCs.

### Ex Situ Spectroscopy Measurements

Optical absorption spectra were collected on a Varian Cary 300 UV–vis absorption spectrophotometer, while PL spectra were recorded by a Varian Cary Eclipse spectrophotometer using an excitation wavelength at 370 nm for all of the samples. The NC solutions were diluted in toluene in quartz cuvettes (path length = 1 cm) to a maximum optical density below 1.0, to minimize self-absorption effects. The time-resolved PL measurements were performed on an Edinburgh FLS900 fluorescence spectrometer equipped with a time-correlated single photon counting unit coupled with an Edinburgh Instruments EPL-510 pulsed laser diodes (λ_exc_ = 372 nm, pulse width = 68.8 ps, and λ_exc_ = 508.2 nm, pulse width = 177.0 ps). Data analysis was carried out using DecayFit software.

### In Situ Spectroscopy Measurements

The in situ ABS and PL spectra were collected directly from the reaction medium via a transmission dip probe (Anglia Instruments, fiber optic immersion probe AIFDP-12UV200600-2-SS-VHT). The signal was collected with an AvaSpec-2048 spectrophotometer controlled through AvaSoft 8 software, version 8.14. All spectra were recorded with a time interval of 5 s, integration time 2 ms, 250 averages per spectrum, and smoothing set to 6 pixels. The light source used for the ABS measurements was an AvaLight-DH-S-BAL, while the light source used for the PL measurements was a Thorlabs adjustable-power 385 nm LED. Data analysis was performed using custom Python scripts developed internally.

### X-ray Diffraction

XRD patterns were collected on a PANanalytical Empyrean X-ray diffractometer, equipped with a 1.8 kW Cu Kα ceramic X-ray tube, operating at 45 kV and 40 mA, a PIXcel3D 2 × 2 area detector, and operating in a parallel beam–symmetric reflection geometry. All of the patterns were acquired at room temperature under ambient conditions. Samples were prepared by first drying the colloidal NC solutions to redisperse them in 50 μL of hexane. This very dense NC suspension was then mixed with fumed silica and dried in place to minimize preferential orientation of the NCs. Finally, the as-obtained powder was deposited on a zero-diffraction silicon substrate to proceed with the measurements.

### Transmission Electron Microscopy

Bright-field TEM images of the samples were acquired with a JEOL-1400Plus transmission electron microscope operating at an acceleration voltage of 100 kV. Samples were prepared by drop casting diluted NCs solutions onto carbon-film-coated 200 mesh copper grids.

### Scanning Transmission Electron Microscopy

HRSTEM images were acquired on an aberration-corrected ThermoFisher Spectra 30-300 S/TEM operated at 300 kV. Samples were prepared by drop-casting diluted solutions of NCs onto ultrathin carbon film-coated 400 mesh lacey carbon copper grids. Atomic resolution images were acquired on a high-angle annular dark field (HAADF) detector with a current of 30 pA and a beam convergence semiangle of 25 mrad. Compositional maps were acquired using Velox, with a probe current of ∼150 pA and rapid raster scanning of the beam. The EDX signal was collected by a Dual-X system comprising two detectors, one on either side of the sample, for a total acquisition solid angle of 1.76 sr.

### Computational Modeling

First-principle electronic structure calculations were performed within the framework of the density functional theory (DFT) formalism^41,42^ as employed in the Vienna Ab-initio Simulation Package (VASP).^43^ The projected-augmented-wave (PAW)^44^ formalism has been implemented throughout the geometry optimization and corresponding electronic structure calculations, while the generalized gradient approximation (GGA) was employed for the exchange correlation functional.^45^ For the bulk and surface geometry optimization, a converged plane wave basis set with a 450 eV energy cutoff with 5 × 5 × 5 and 5 × 5 × 1 Monkhorst Pack K-points was applied.^46^ DOS calculations were performed after performing a structure relaxation step, with convergency minimum-energy criteria being Hellman–Feynman force < 0.01 eV/Å.

## Supporting Information Available

Morphological and compositional analysis of the pristine CsPbCl_3_ NCs by TEM; size distribution histograms of pristine CsPbCl_3_ NCs and of CsPbCl_3_ NCs reacted with increasing amounts of PbI_2_; experimental data on Cl → I halide exchange on DDACl-capped CsPbCl_3_ NCs using various halide sources; additional optical and XRD data on experiments performed using PbI_2_ as halide source (including tables on reactions conditions and NC compositions); morphological and compositional analysis (by TEM) of partially exchanged CsPbCl_3_ NCs with different halides ratios; additional HAADF-STEM analyses of individual CsPbCl_3_ NCs covered by a CsI surface layer; in situ PL spectra of a solution of pristine CsPbCl_3_ NCs after the addition of an OA/OLA/ODE solution; absorption spectrum of the PbI_2_ precursor solution; binary phase diagram conceptualizing the formation of miscibility gaps in mixed-halide LHP systems (PDF)
