# Supplementary material for: CsPbCl3 → CsPbI3 Exchange in Perovskite Nanocrystals Proceeds through a Jump-the-Gap Reaction Mechanism
Source: J Am Chem Soc. 2023 Sep 11;145(37):20442–50. doi: 10.1021/jacs.3c06214 (PMC10515632; doi:10.1021/jacs.3c06214)
Supplement: Supplementary file 1 — ja3c06214_si_001.pdf [file ja3c06214_si_001.pdf]

Supporting Information for

$CsPbCl_3 \rightarrow CsPbI_3$ Exchange in Perovskite Nanocrystals Proceeds Through a Jump-the-Gap Reaction Mechanism

Nikolaos Livakas[a, b], Stefano Toso[a*], Yurii P. Ivanov[c], Tisita Das[d], Sudip Chakraborty[d], Giorgio Divitini[c], Liberato Manna[a*]

[a] Nanochemistry, Istituto Italiano di Tecnologia, Via Morego 30, 16163 Genova, Italy

[b] Dipartimento di Chimica e Chimica Industriale, Università di Genova, 16146 Genova, Italy

[c] Electron Spectroscopy and Nanoscopy, Istituto Italiano di Tecnologia, Via Morego 30, 16163 Genova, Italy

[d] Materials Theory for Energy Scavenging (MATES) Lab, Department of Physics, Harish-Chandra Research Institute (HRI), A CI of Homi Bhabha National Institute (HBNI), Chhatnag Road, Jhunsi, Prayagraj 211019, India.

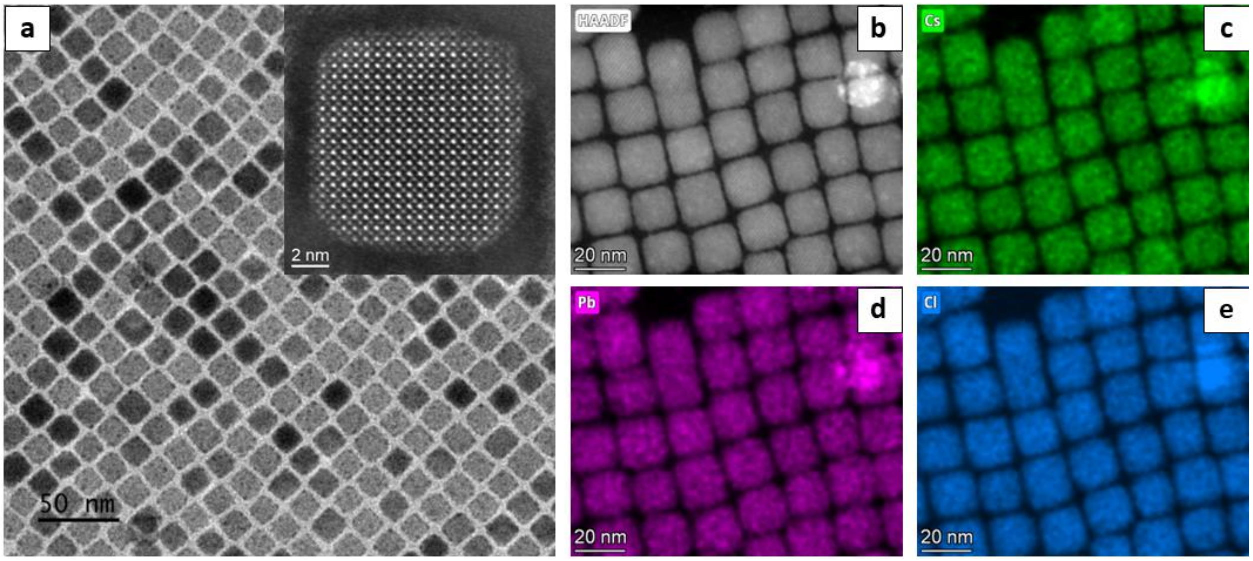

Figure S1. **Morphology and composition of pristine $CsPbCl_3$ NCs**. a) TEM image of as synthesized NCs. Inset: High-resolution HAADF-STEM image of a single NC. b-e) HAADF-STEM image (b) with the corresponding EDX elemental maps for cesium (c), lead (d), and chloride (e).

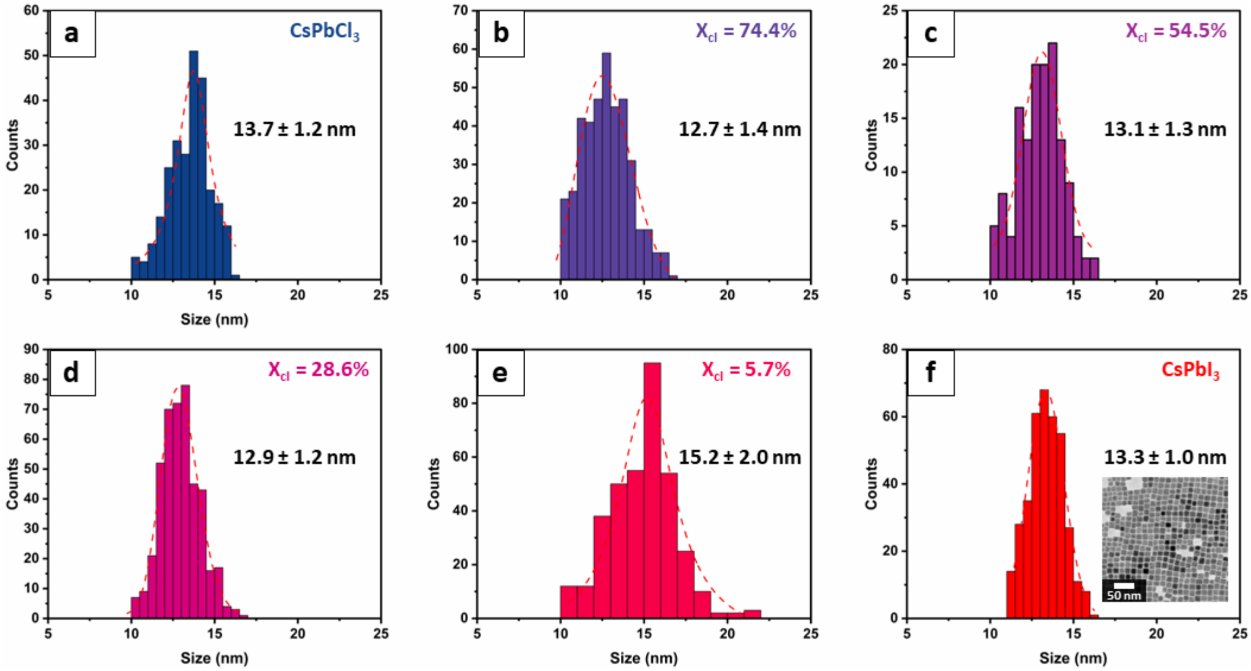

Figure S2. Size distribution histograms. a) Pristine $CsPbCl_3$ NCs. b-e) $CsPbCl_3$ NCs reacted with increasing amounts of PbI_2 from partial to nearly full conversion to $CsPbI_3$ NCs. f) Pristine $CsPbI_3$ NCs. The NCs size distribution histograms were estimated using the Ilastik[1] software.

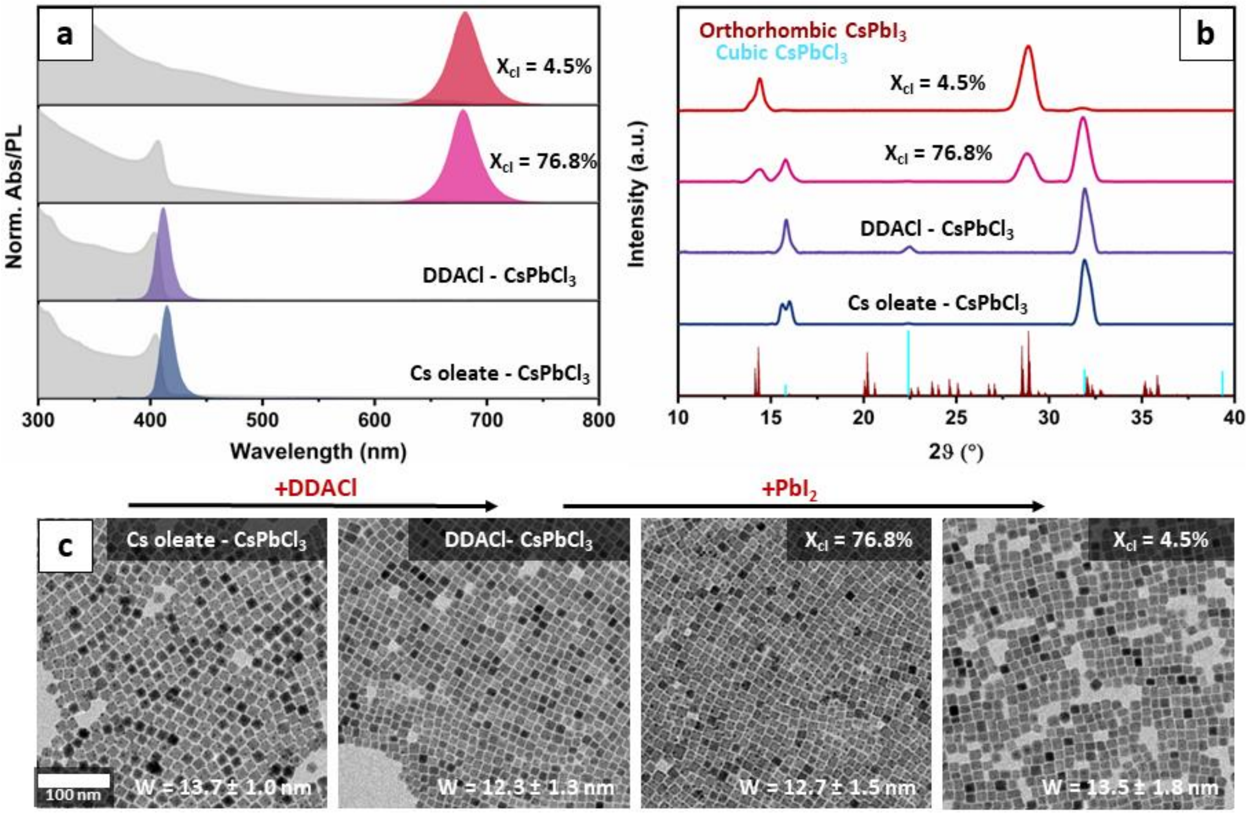

Figure S3. Cl→I halide exchange on DDACl-capped $CsPbCl_3$ NCs using PbI_2 as the halide source. a) ABS (grey) and PL (colored) spectra of pristine and exchanged $CsPbCl_3$ NCs with increasing amounts of PbI_2. b) XRD patterns and (c) BF-TEM images of the same samples.

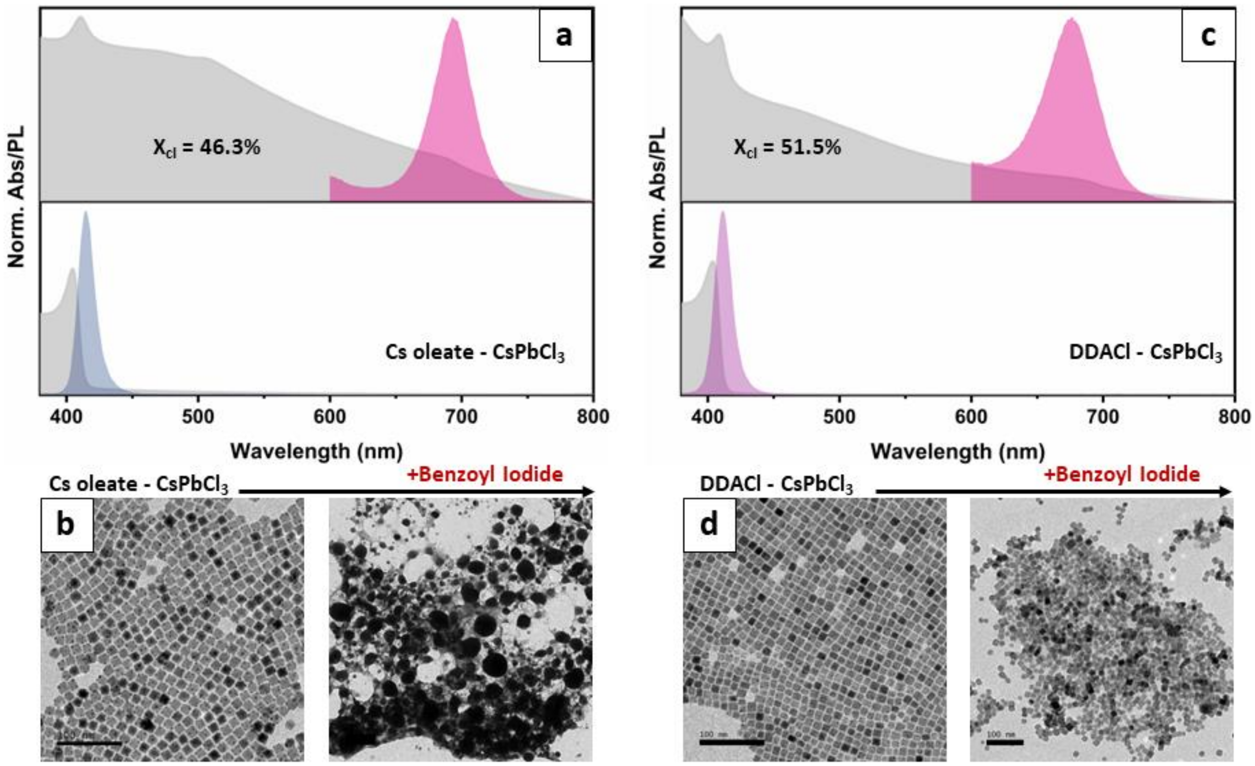

Figure S4. Cl→I exchange on oleate- and DDACl-capped $CsPbCl_3$ NCs using benzoyl iodide as the halide source. a) ABS (grey) and PL (colored) spectra, and b) TEM images of both pristine and exchanged oleate capped $CsPbCl_3$ NCs. c,d) Analogous exchange and characterization techniques applied for DDACl capped $CsPbCl_3$ NCs.

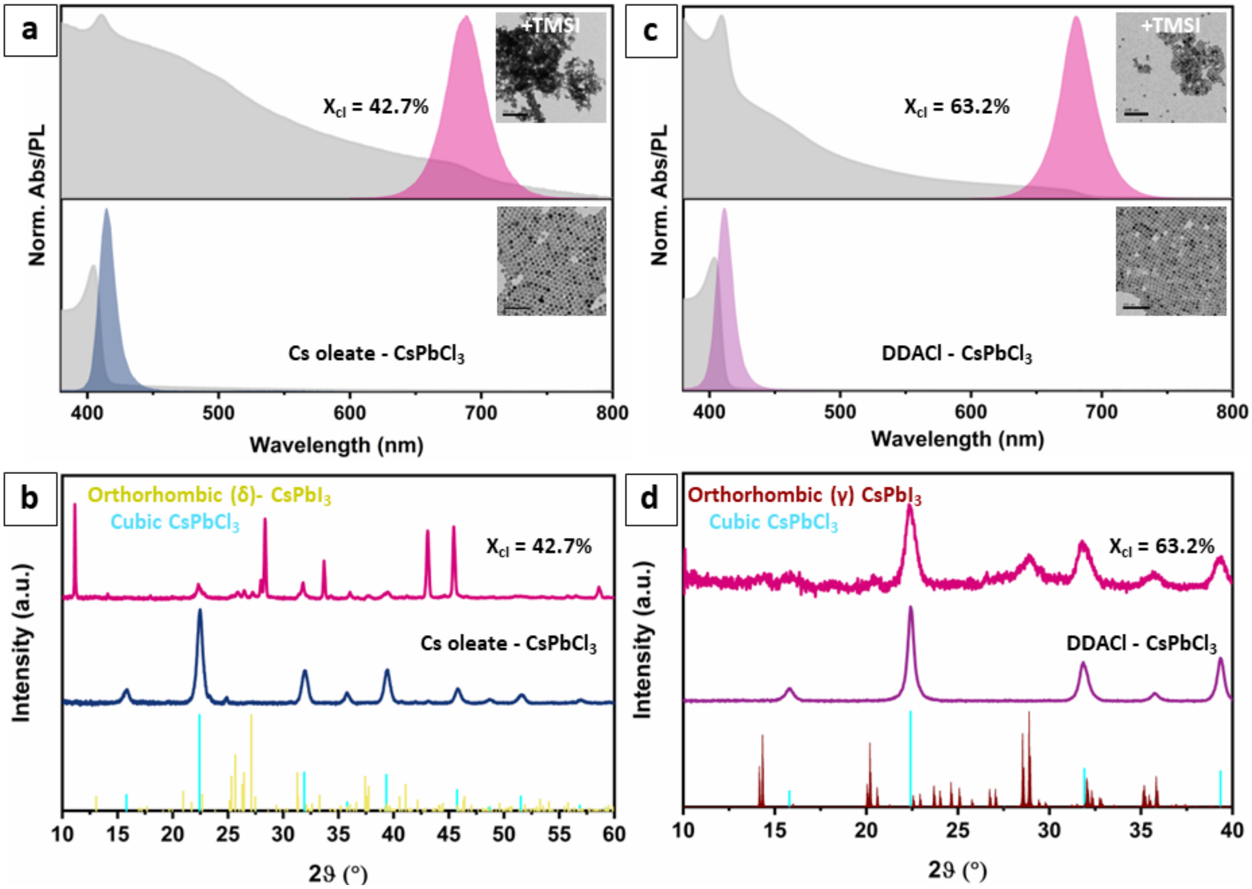

Figure S5. Cl→I exchange on oleate- and DDACl-capped $CsPbCl_3$ NCs using TMSI as the halide source. a) ABS (grey) and PL (colored) spectra, TEM images (insets), and b) XRD patterns of both pristine and exchanged oleate capped $CsPbCl_3$ NCs. The anion exchanged sample transformed to the non-perovskite δ-$CsPbI_3$ polymorph during the XRD measurement, due to its intrinsic instability and the exposure to air and moisture. The remaining sharp peaks not compatible with the δ-$CsPbI_3$ are attributed to the minary compounds CsI and PbI_2. c,d) Similar experiments performed on DDACl capped $CsPbCl_3$ NCs.

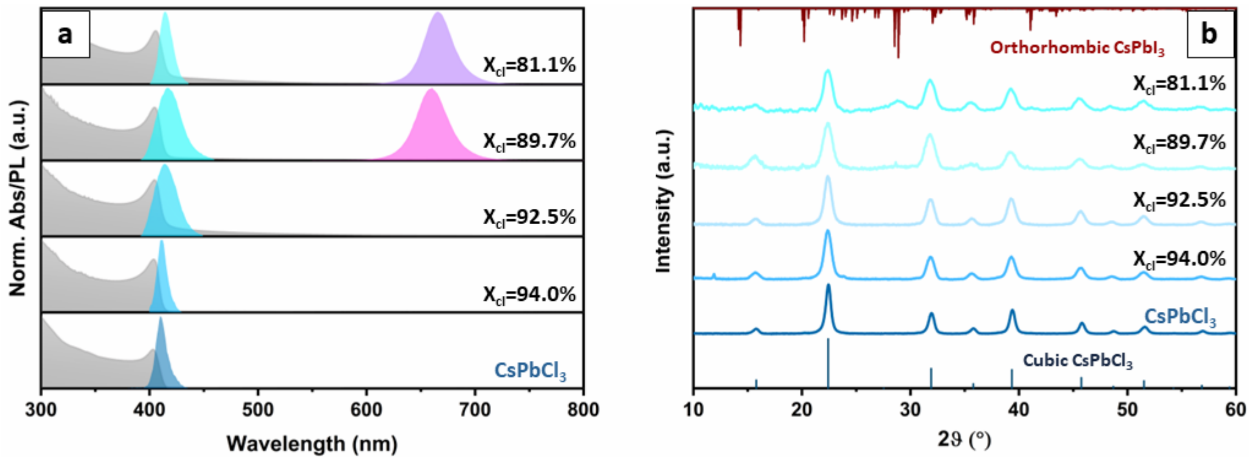

Figure S6. Multiple halide exchanges inducing several halides ratios (expressed by X_{Cl}) before and after the appearance of the $CsPbI_3$ phase. a) ABS (grey) and PL (colored) spectra of pure-halide $CsPbCl_3$ and I^- doped $CsPbCl_3$ NCs, and of exchanged $CsPbCl_3$ NCs with increasing amounts of PbI_2. b) XRD patterns of the same samples.

Table S1. Conditions for the exchange experiments discussed in Figure 1. All exchanges were performed on 6 ml aliquots of $CsPbCl_3$ NCs with constant concentration (see Methods). The concentration of Cl in the pristine sample was estimated indirectly based on ICP-OES to determine lead concentration ([Pb] = 6.3 mM), and assuming a stoichiometric ratio of Pb:Cl=1:3 ([Cl] = 18.9 mM). The actual concentration of Cl after each addition was therefore calculated based on the starting volume of the sample and the volume of the PbI_2 stock solution added for the exchange. The concentration of I in the exchanged solution was calculated based on the moles of PbI_2 in the stock solution and the volume of stock solution added for each experiment. The final halide composition of the exchanged NCs was measured experimentally post-exchange by EDX, as detailed in the Main Text.

PbI_2 stock solution added (mL)	Chloride concentration in solution (mM)	Iodide concentration in solution (mM)	EDX (X_{cl} %)
0.25	18.1	4.0	94.0
0.50	17.4	7.7	92.5
1.00	16.2	14.3	89.7
1.10	16.0	15.5	81.1
1.30	15.5	17.8	74.4
1.70	14.7	22.1	54.5
2.00	14.2	25.0	28.6
6.00	9.5	50.0	5.7

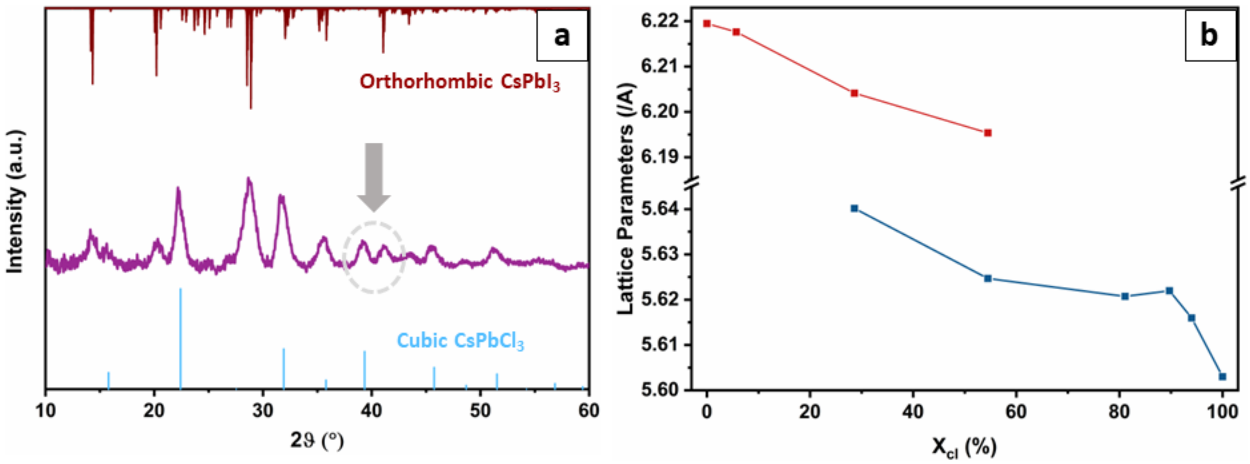

Figure S7. Lattice parameters extraction from $CsPbCl_3$ and $CsPbI_3$ NCs. a) XRD pattern of one of the exchanged $CsPbCl_3$ NCs (X_{cl} = 54.5%). Circled in grey are the (211) pseudocubic peaks of both $CsPbCl_3$ and $CsPbI_3$ NCs, which were chosen for the extraction of lattice parameters because they were the only high-angle peaks with limited overlap with other reflections. In this case, we preferred not to perform a full-profile (e.g., Rietveld or Le Bail) because the overlap of many reflections within each experimental peak, broadened by finite-size effects, would make the fit converge to unreliable results. b) Lattice parameters as a function of x_{Cl} (tuned via the amount of added PbI_2, and measured experimentally after the exchange). The analysis indicates that some iodide is incorporated within the volume of $CsPbCl_3$ NCs, and vice versa. The samples used for the analysis are presented in Figures 1 and S6. The sample with X_{cl} = 92.5% was not included in the plot (b) to avoid the overlap of the experimental points with the X_{cl} = 94% sample, as their lattice parameters are very close.

Table S2. Average NCs compositions estimated by Vegard's law. Compositions of I-doped $CsPbCl_3$ NCs and Cl-doped $CsPbI_3$ NCs estimated by applying the Vegard's law[2] [$d_{mixed\text{-}halide\ NCs} = x_{Cl} \cdot d_{pure\ Cl\ NCs} + (1 - x_{Cl}) \cdot d_{pure\ I\ NCs}$].[2] For the purpose of the analysis, the structure of $CsPbI_3$ was approximated as cubic. We chose the lattice constants of as-synthesized pure-halide NCs as reference values to minimize errors related to the small lattice expansion of NCs if compared to their relative bulk references.

Sample composition (x_{Cl}%)	$CsPbCl_3$ lattice parameter (Å)	$CsPbI_3$ lattice parameter (Å)	I^- fraction in $CsPbCl_3$ NCs (%)	Cl^- fraction in $CsPbI_3$ NCs (%)
100 (Pure $CsPbCl_3$)	5.603	-	0	-
94	5.616	-	2.10	-
92.5	5.616	-	2.12	
89.7	5.622	-	3.08	-
81.1	5.621	-	2.88	-
74.4	5.632	-	4.63	-
54.5	5.625	6.195	3.52	3.92
28.6	5.640	6.204	6.03	2.49
5.7	-	6.218		0.30
0 (Pure $CsPbI_3$)	-	6.220	-	0

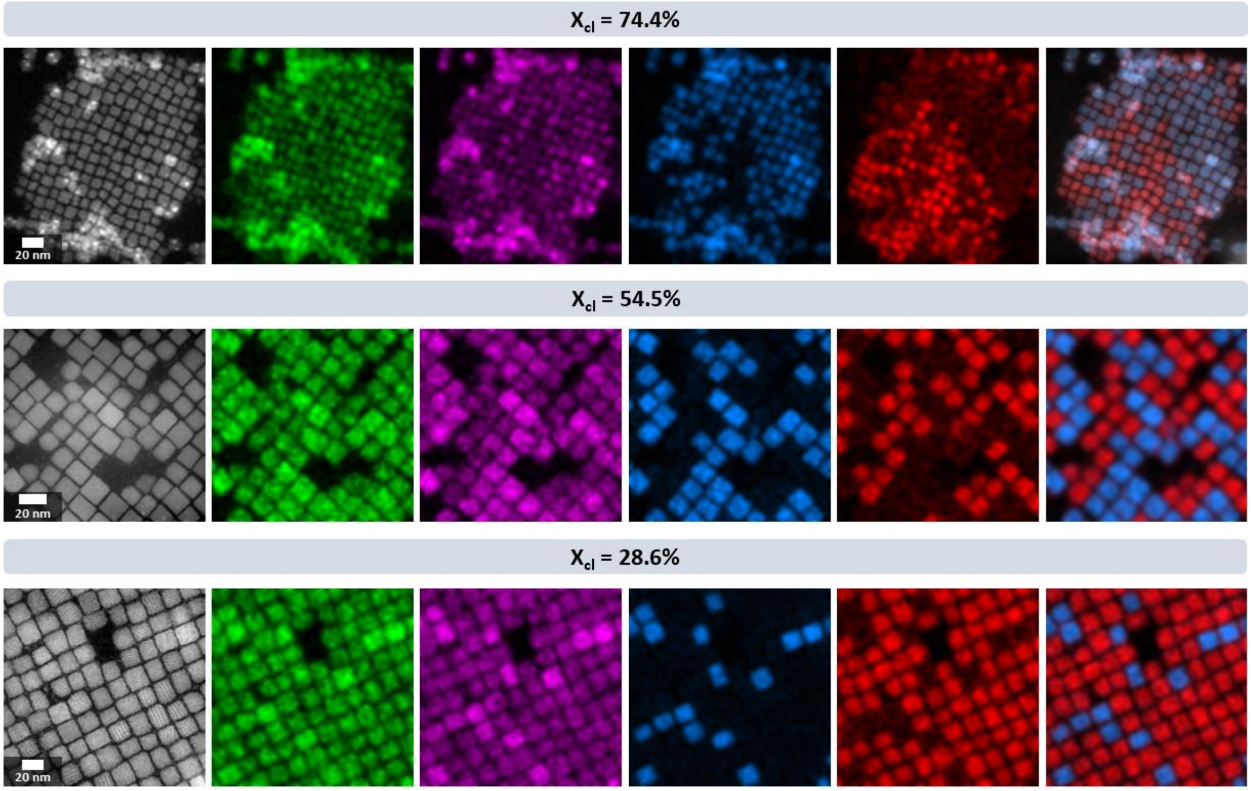

Figure S8. Morphology and composition of partially exchanged $CsPbCl_3$ NCs upon different halides ratios (i.e. various X_{Cl} %). HAADF-STEM image with the respective EDX mapping for each partially exchanged case (X_{Cl} = 28.6% at the bottom row, X_{Cl} = 54.5% at the middle row, and X_{Cl} = 74.4% at the upper row). EDX mapping refers to Cs (green), Pb (purple), Cl (blue), and I (red).

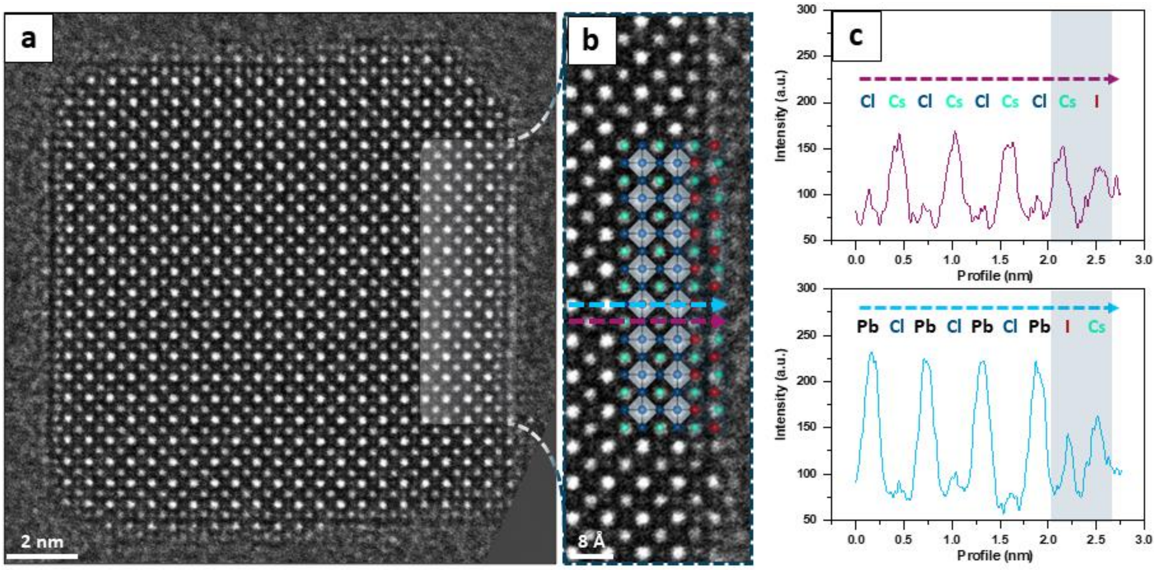

Figure S9. Intensity profile analysis of Figure 2f. a) Reproduction of Figure 2f. b) Magnified area of the $CsPbCl_3$ NC surface, indicating the presence of a Cs-I surface layer (Cs^+ = Green, Cl^- = blue, I^- = red, and $[PbCl_6]^{4-}$ octahedra = light blue). The dashed arrows indicate the atomic planes chosen to extract the cross-sections shown in panel (c). c) Cross-section of the intensity profiles along both Cs-Cl (upper panel) and Pb-Cl (lower panel) planes, indicated in panel (b) by the purple and cyan colored arrows, respectively. The cross sections demonstrate an increase of signal intensity compared to the corresponding halide positions in the NCs bulk, which is attributed to a Cl→I replacement limited to the NC surface.

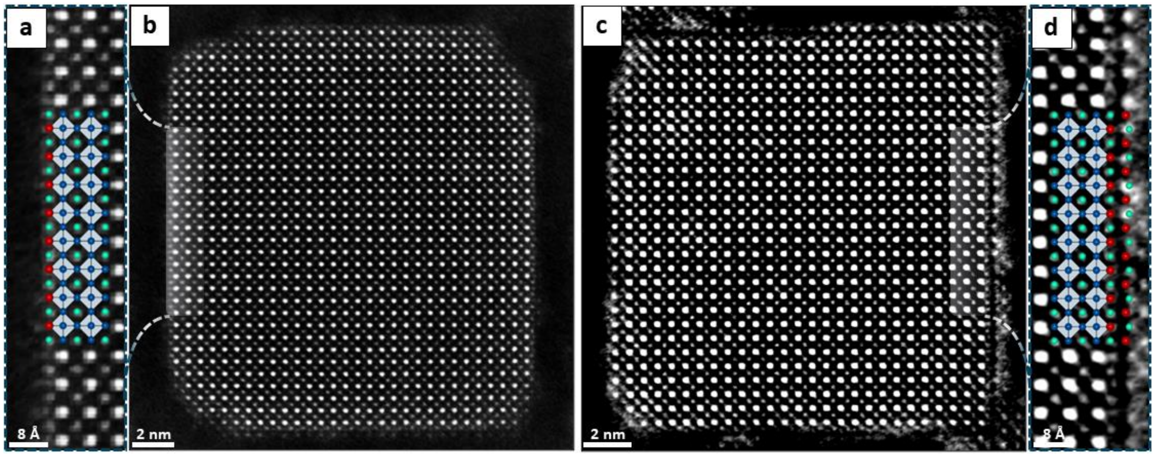

Figure S10. Examples of $CsPbCl_3$ NCs showing the formation of Cs-I surface layers. b, c) Atomic-resolution images of $CsPbCl_3$ NCs after partial exchange, with a, d) their magnified areas of the NCs' surface, respectively. Notably, in the first case (panel a, b), only one Cs-I plane is observed on the $CsPbCl_3$ NC, whereas in the latter one (panel c, d), also a second Cs-I plane shifted by half-cell is preset, giving rise to a structural motif characteristic of the all-inorganic Ruddlesden-Popper phase $Cs_2PbCl_2I_2$.

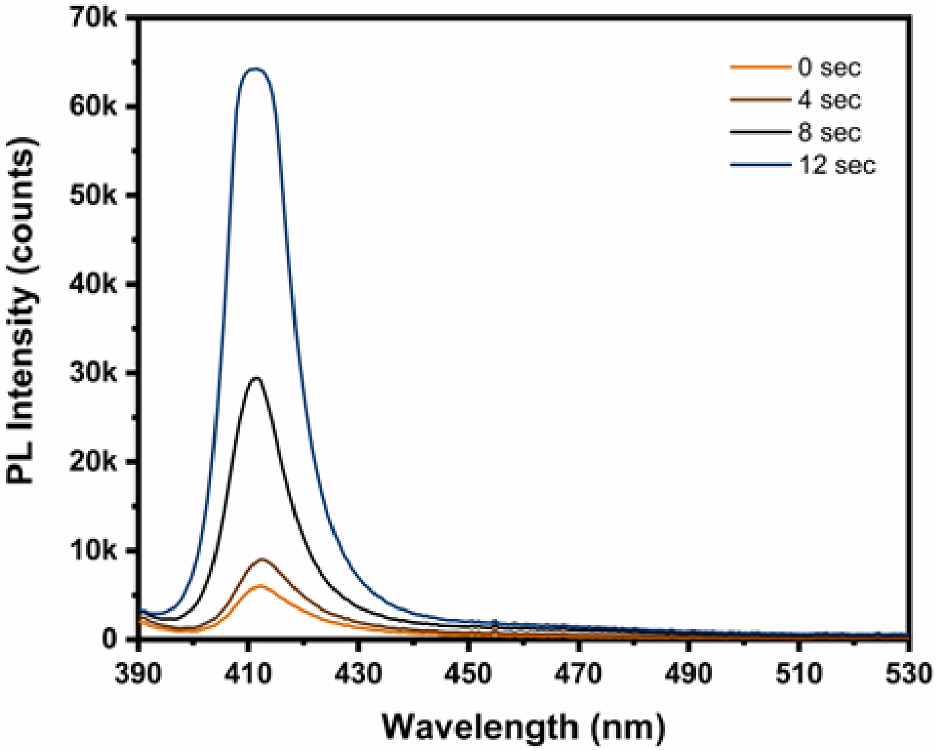

Figure S11. In-situ PL spectra. The spectra were collected every 4 seconds starting after the addition of OA/OLA/ODE in a solution of pristine $CsPbCl_3$ NCs. The incorporation of OA/OLA leads to a fast increase in the PL intensity of the NCs immediately after the injection.

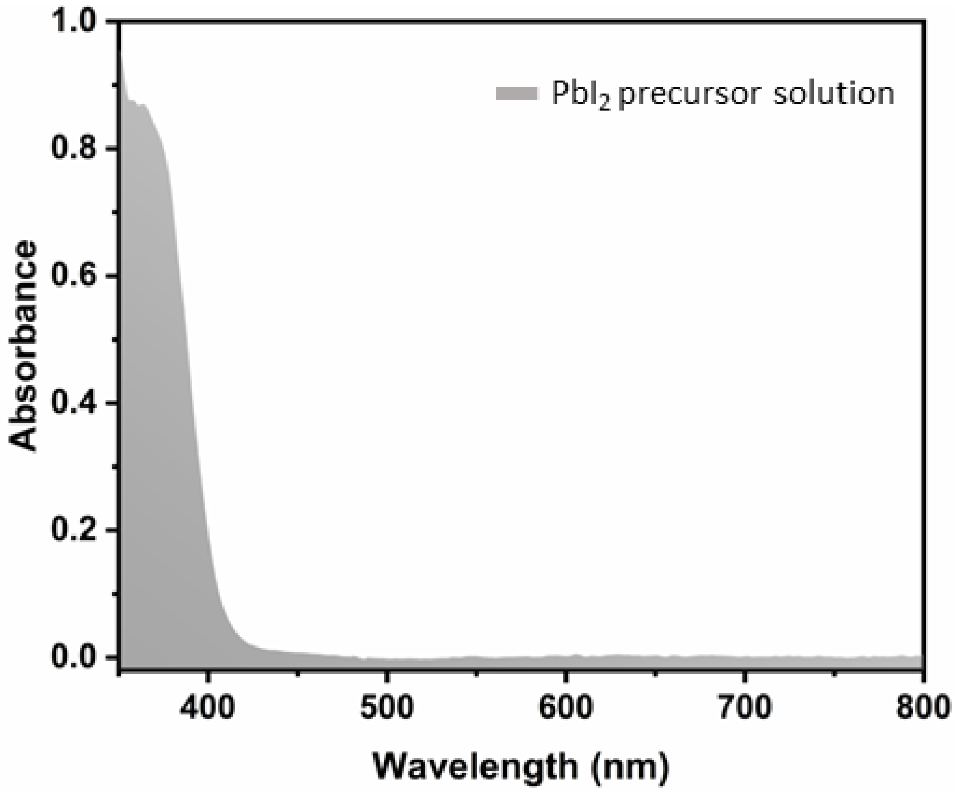

Figure S12. Absorption spectrum of the PbI_2 precursor solution.

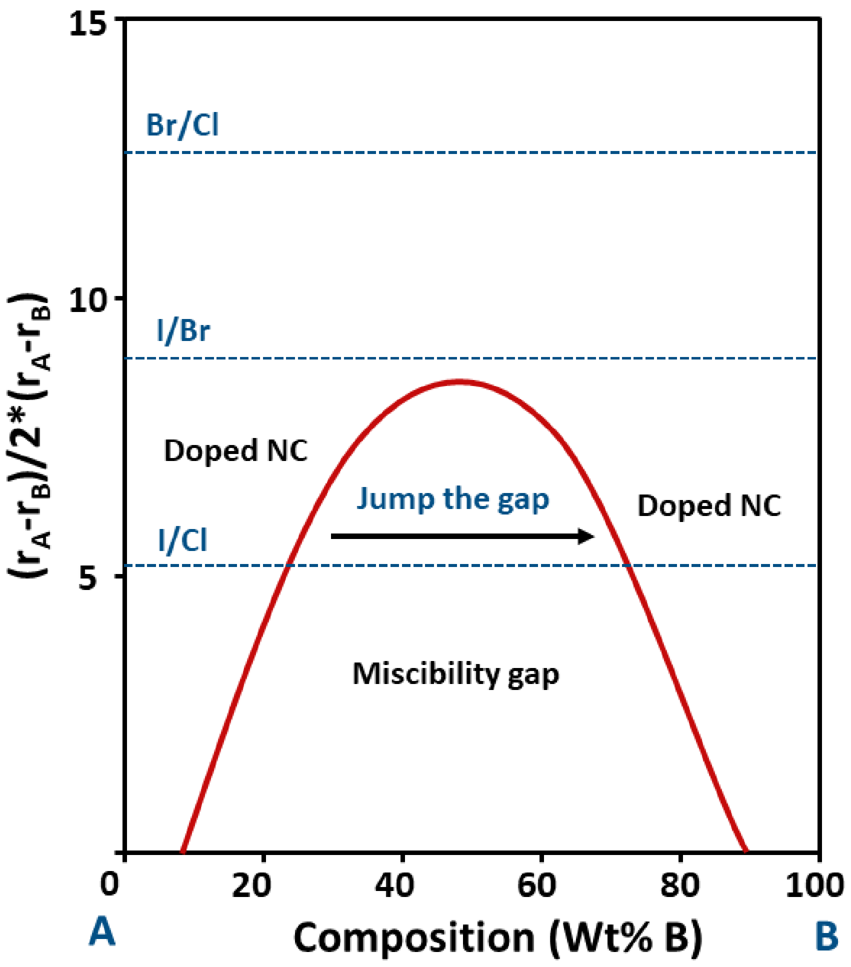

Figure S13. Miscibility gap represented in a halide-ratio / relative ionic radii phase diagram. The miscibility of two $CsPb(A)_3$ and $CsPb(B)_3$ lead halides perovskites (where A, B = Cl, Br, I and A ≠ B) can be represented in binary phase diagram where the vertical coordinate indicates the of A and B in ionic radii (higher *y* values = closer radii). When the ionic radii difference is small (high *y*-values), the system can form a continuous solid solution (i.e., Br/Cl and I/Br pairs). However, when the difference is substantial (low *y*-values), a miscibility gap is formed, and each individual $CsPbX_3$ compound can only accept a limited concentration of the other halide (as is the case of Cl/I) due to the significant lattice strain this induces. In such case, an increase in the concentration of the second halide will force the system to leap over the miscibility gap, as reported in this work.

References

(1) Berg, S.; Kutra, D.; Kroeger, T.; Straehle, C. N.; Kausler, B. X.; Haubold, C.; Schiegg, M.; Ales, J.; Beier, T.; Rudy, M.; Eren, K.; Cervantes, J. I.; Xu, B.; Beuttenmueller, F.; Wolny, A.; Zhang, C.; Koethe, U.; Hamprecht, F. A.; Kreshuk, A. Ilastik: Interactive Machine Learning for (Bio)Image Analysis. *Nat Methods* **2019**, *16* (12), 1226–1232.

(2) Vegard, L. Die Konstitution Der Mischkristalle Und Die Raumfüllung Der Atome. *Zeitschrift für Physik* **1921**, *5* (1), 17–26.
